# Recommendations for the Design of Serious Games in Neurodegenerative Diseases

**DOI:** 10.3389/fnagi.2018.00013

**Published:** 2018-02-02

**Authors:** Grégory Ben-Sadoun, Valeria Manera, Julian Alvarez, Guillaume Sacco, Philippe Robert

**Affiliations:** ^1^CoBTeK Lab – Cognition Behaviour Technology, Université Côte d’Azur, Nice, France; ^2^Association Innovation Alzheimer – Autism – Affect, Nice, France; ^3^EA 2445 – DeVisu, Université de Valenciennes, Valenciennes, France; ^4^Play Research Lab – Serre Numérique, CCI Nord GH, Valenciennes, France; ^5^SSR Post-AVC, Pôle Réhabillitation Autonomie Vieillissement, CHU de Nice, Université Côte d’Azur, Nice, France; ^6^Centre Mémoire de Ressources et de Recherche, CHU de Nice, Université Côte d’Azur, Nice, France

**Keywords:** serious games, ergonomic criteria, game design Alzheimer Disease, mild cognitive impairment

## Abstract

The use of Serious Games (SG) in the health domain is expanding. In the field of Neurodegenerative Diseases (ND) such as Alzheimer’s Disease, SG are currently employed to provide alternative solutions for patients’ treatment, stimulation, and rehabilitation. The design of SG for people with ND implies collaborations between professionals in ND and professionals in SG design. As the field is quite young, professionals specialized in both ND and SG are still rare, and recommendations for the design of SG for people with ND are still missing. This perspective paper aims to provide recommendations in terms of ergonomic choices for the design of SG aiming at stimulating people with ND, starting from the existing SG already tested in this population: “MINWii”, “Kitchen and Cooking”, and “X-Torp”. We propose to rely on nine ergonomic criteria: eight ergonomic criteria inspired by works in the domain of office automation: Compatibility, Guidance, Workload, Adaptability, Consistency, Significance of codes, Explicit control and Error management; and one ergonomic criterion related to videogame: the game rules. Perspectives derived from this proposal are also discussed.

## Introduction

Due to population aging, the number of people with neurodegenerative disease (ND) leading to dementia, a decline in mental ability that interferes with activities of daily living, is predicted to escalate in the next 50 years (Alzheimer’s Disease International, 2014). Dementia can result from different causes, the most common being Alzheimer’s disease (AD). It is often preceded by a pre-dementia stage, known as Mild Cognitive Impairment (MCI), characterized by a cognitive decline greater than expected for an individual’s age, which, however, does not interfere notably with activities of daily living (Petersen et al., 2009). Depending on the etiology and the disease’s stage, dementia can be characterized by cognitive, behavioral, motor, and/or functional symptoms. From a therapeutic point of view, much research aims to modify the course of the disease or to reduce the impact of the clinical symptoms through non-pharmacological interventions. Based on the Cognitive-Enrichment hypothesis (Hertzog et al., 2008), effective non-pharmacological approaches include cognitive stimulations, physical activities, social engagement, positive affects, and reduction of psychological distress.

The idea to use video games (VG) as Enriched Environments (interventions targeting simultaneously multiple domains) is quite recent (Anderson-Hanley et al., 2012; Maillot et al., 2012). It derives from the acknowledgment that VG are intrinsically entertaining and can stimulate cognitive functions (Powers et al., 2013; Toril et al., 2014), involve physical exercise, social engagement, and favor positive emotions, thus leading to synergistic effects on cognitive functions and neuroplasticity (Anderson-Hanley et al., 2012; Maillot et al., 2012). The cognitive decline found in older adults with AD or MCI questions the usability of the VG designed for “healthy players” (Ben-Sadoun et al., 2015). For this reason, there is a recent interest among professionals to design specific VG (called Serious Games [SG]) adapted to these populations (Robert et al., 2014; Manera et al., 2017), in order to slow down the cognitive decline associated with normal and pathological aging (Mader et al., 2012; Anguera et al., 2013; Kim et al., 2016).

At present, professionals working on ND mostly have a lack of theoretical knowledge concerning VG, SG and, more generally, the principles of human-machine interactions. For instance, “SG” is sometimes employed as an umbrella term to include neuropsychological training software, VG and SG (McCallum and Boletsis, 2013). A VG is a “*A mental contest played with a computer in accordance to specific rules designed for amusement, entertainment or victory purposes, through the notion of challenge”* (Zyda, 2005). Following Alvarez (2007), a SG can be distinguished from a VG because the initial goal of its designer is to integrate a pedagogic scenario into the game scenario. Finally, neuropsychological software (which is not VG or SG) does not embed rules meant to promote entertainment.

In parallel, professionals developing VG have mostly a lack of knowledge of the cognitive and behavioral consequences of ND. They often overestimate the abilities of ND patients, considering them as comparable to those of healthy elderly. Consequently, the VG are mostly tested only on healthy elderly. This poses serious problems in terms of testing the real product usability, which depends, for instance, on ‘*the degree to which the product can be used by the target users to achieve defined goals with efficiency, efficacy and satisfaction in a specified context of use* (ISO 9241-11:1998, 1998).

How it is possible to help professionals in the SG design to ensure optimized product usability? As suggested by the standard ISO 9241-11, it is important to suggest ergonomic criteria to adopt. Bastien and Scapin (1992) described eight main ergonomic criteria to improve the interaction between humans and new Information and Communication Technologies (ICT). However, these criteria did not include any ‘Game’ component, which is typical of SG.

This perspective paper aims to provide recommendations in terms of ergonomic choices for the design of SG to stimulate people with ND, starting from existing SG already tested in this population. Specifically, we will employ the eight ergonomic criteria proposed by Bastien and Scapin (1992) to make specific recommendations for the design of SG for ND, and we will delineate some perspectives on how to enrich those criteria thanks to models derived from SG principles.

## Serious Games for People with MCI and AD

If we stick to the SG definition described in the previous paragraph (Alvarez, 2007), to the best of our knowledge, there are only three SG in the scientific literature designed for stimulation and tested on our target population (people with MCI or AD from early to moderate stages). The first, MINWii (on Nintendo^®^ Wii^TM^, Benveniste et al., 2012) is a music SG in which the player plays on a virtual keyboard well-known French songs. The goal is to increase positive emotions. The second, Kitchen and Cooking (on tablet, Manera et al., 2015) is a cooking SG in which the player, following a recipe, looks for the ingredients in a virtual kitchen and plans the actions necessary to complete the recipe. The goal is to stimulate cognitive functions and positive emotions. The third, X-Torp (on Microsoft^®^ Kinect^TM^; Ben-Sadoun, 2016; Ben-Sadoun et al., 2016) is an action-adventure SG in which the player takes the command of a submarine, and following a story plot, fights against virtual ships and accomplishes short missions (mini-puzzle games). The goal is to stimulate cognitive and physical activity, and favor positive emotions.

### Compatibility

This criterion (**Table 1**) highlights the importance of relating ICT to the characteristics of the users (Bastien and Scapin, 1992). According to Karwowski (2000), it “should be considered at all levels, including physical, perceptual, cognitive, emotional, social, organizational, and environmental considerations.” People with MCI and AD-related ND suffer from physical, behavioral, and cognitive disorders (see below). In addition, due to their age, they are frequently novices in the use of ICTs and are not familiar with the current VG ergonomic features (e.g., color or symbols codes). Finally, their motivations to play may be different from those of young people and of healthy elderly. Although the evaluation of the motivation to play is of crucial importance to explain technology adhesion, to our knowledge, no study addressed this question so far in elderly. Specific models, such as the theory of self-determination of Ryan and Deci (2000) already used for the young novices (Ryan et al., 2006) may provide guidelines for this research.

**Table 1:** Summary of selected ergonomic criteria, of the recommendations and perspectives for the design of Serious Games (SG).

Criteria	Definition	Main recommendations	Perspectives
Compatibility	Match between users’ characteristics (e.g., cognitions, skills, age, expectations) and system characteristics (including the user interface)	- Take into account target players’ cognitive, physical, behavioral and functional characteristics - Test the SG on this exact population, to evaluate the ergonomic choices concerning the other criteria	- Evaluate the motivations to play VG and SG for people with MCI and AD
Guidance	Means available to advise, orient, inform and guide the users throughout their interactions with a ICT system to ensure that they always know where they are and what they can do	- Reconcile the need of guidance and of providing a challenge but... - Permanently provide real time guidance - Provide constant feedback on the game progression - Take advantage of educational mediators - Group the informations following their common sense on the interface - Display possible actions (commands) associated with simple and readable feedback	
Workload	All interface elements available to reduce of the users’ perceptual or cognitive load, and to increase the dialogue efficiency	- Limit the number of game commands (max 6) - Reduce motor actions that monopolize attentional resources - Reduce the number of information on the interface to those which are necessary	
Adaptability	System capacity to behave contextually and according to the users’ needs and preferences	- Limit flexibility to favor a linear relationship between goals and actions - Integrate different difficulty levels for the cognitive and/or physical challenges for a stimulation of progressive intensity, keeping constant guidance parameters	- Use difficulty levels to create different SG versions for different pathology profiles (e.g., amnesic MCI, non-amnesic MCI, AD)
Consistency	The way interface design choices (e.g., codes, naming, location, formats, procedures) are maintained in similar contexts, and are different when applied to different contexts	- Follow Bastien and Scapin (1992)’s recommendations	
Significance of codes	The relationship between a term/sign and its reference. Codes and names are significant when there is a semantic relationship between such codes and the items or actions they refer to	- Determine which commands, images, messages, and game scenarios are “familiar enough” to be understood, memorized and recognized by the target players - Select explicit images - Employ selection commands with clear and concise text (“Validate”) - Choose meaningful command modes - Avoid classical VG codes and symbols (unknown to the target players)	
Explicit control	System processing of explicit user actions, and the control users have on the processing of their actions by the system	- Follow Bastien and Scapin (1992)’s recommendations	
Error management	Means available to prevent or reduce errors (e.g., invalid data entry, incorrect command syntax), and to recover from them when they occur	- Follow Bastien and Scapin (1992)’s recommendations	
Game rules	General and local player’s goals, together with the means of action and degrees of freedom available in the virtual world	- Define the game rules based on the challenges (gameplay metabrics) of Djaouti et al. (2008) - Choose the challenges adapted to stimulate the cognitive functions targeted by the SG - Do not employ more than 2 challenges simultaneously - Avoid the “WRITE” action, in which the user needs to enter an alphanumeric string to respond	- Establish correspondences between game challenges and cognitive functions - Enrich the model by including social challenges and control modes (sedentary, physical)

### Guidance

Guidance criterion (**Table 1**) refers to the means available to lead the users to making specific actions within the context and providing information on the actual state. This “prompting” helps the player evolving in a game where results uncertainty is a necessary ingredient to guarantee the presence of a challenge (i.e., the player is not certain of succeeding; Caillois, 1958; Malone, 1980). Studies on MINWii (Benveniste et al., 2012) and X-Torp (Ben-Sadoun, 2016) showed that the original SG versions required modifications after being tested due to an incorrect parameterization of this criterion with respect to some characteristics of the target population. AD is characterized by the presence of apathy – a disorder of motivation and self-initiated activities (Marin, 1990; Mulin et al., 2011), as well as memory impairment, with a reduced ability to learn and recall new knowledge (Karlsson et al., 2002; Camus et al., 2003). Due to these features, it is important to guide the player more than possible. The first X-Torp version did not prompt enough the player on the actions to be performed to advance in the game scenario, had too many degrees of freedom (e.g., missions could be accomplished in any order), and did not provide enough feedback on the game progression. After being tested on the target population, the SG was improved by providing a permanent guidance on the actions to be performed (e.g., “move there to continue”; “in this mission you need to …”), reducing the number of alternatives (e.g., missions accomplished in a predefined order), and providing permanent feedback on the scenario progression (e.g., “mission number …”). To reinforce guidance, these SG included pedagogical mediators, either humans (in MINWii and X-Torp) or digital (Kitchen and cooking), which are necessary, following Alvarez et al. (2017), to (1) promote the assimilation of SG rules for all the target users (How to play), and (2) to help them understanding the SG final goals (Why to play). The goal is to avoid that the player gets stuck for too long, which could result in game disengagement.

Guidance criterion also refers to the importance of distinguishing different kinds of information by grouping or formatting them in a meaningful way. For instance, in the SG X-Torp (Ben-Sadoun, 2016), the information about the player position on the game map are on the top of the screen, while all navigation commands are at the bottom of the screen. Finally, guidance refers to the fact that the player should easily understand the possible actions to perform, and have clear and easily legible action feedbacks. For instance, in MINWii (Benveniste et al., 2012), the piano key to be selected was highlighted.

### Workload

The higher the workload (**Table 1**), the more likely it is that the player makes mistakes, and gets tired. Targeting a population with impairments in memory, attention and executive functions, the number of actions to be performed should be low, and the commands should be straightforward and simple to master. For instance, in Kitchen and Cooking (Manera et al., 2015) only two types of actions were possible in the recipe planning stage (selecting an ingredient by touching it, and dragging it up and down the ingredient list to rank its order). In X-Torp, the player had to master seven commands to navigate. This was too complex for the target player to master. The help of the clinician was indispensable to guarantee a correct command understanding and utilization during the game (Ben-Sadoun, 2016). Also, the amount of information displayed on the screen should be limited. To judge if the amount of displayed information is correct for a target population, preliminary usability tests are recommended, in order to identify and eliminate unnecessary or ambiguous information.

### Adaptability

Adaptability (**Table 1**) refers to the system flexibility (i.e., the existence of different means to reach a target), as well as to the possibility to personalize user interfaces based on the user level of experience. While flexibility is an important aspect to guarantee system usability in healthy people with a good ICT expertise (Bastien and Scapin, 1992), we believe that for our target population, flexibility should be minimized in order to obtain a more linear relationship between actions and their goals. We believe it is not necessary to automatically adapt the game guidance parameters to the user experience (e.g., beginner, expert). However, it could be useful to create different levels of difficulty based on the user’s type of cognitive impairment (e.g., amnesic MCI, non-amnesic MCI, AD, mixed dementia), according to the recommendations provided by the compatibility criterion.

### Consistency

As for the guidance criterion, for “consistency” (**Table 1**), we believe it is important to follow the recommendations of Bastien and Scapin (1992). In particular, it is important to be consistent in the localization of the items in the user interface (titles, windows, etc), in the screen formats, in the menu options, and in the format of the instructions and feedback messages.

### Significance of Codes

Significance of codes criterion (**Table 1**) is crucial for our target populations, because impairments in language and semantic memory are common, including troubles in language understanding, sentence repetitions, and difficulty in retrieving semantic knowledge of words and objects (Belin et al., 2006). Therefore, we recommend not to employ the standard codes and denominations used in VG and ICT interfaces (which are likely unfamiliar to people in our target population), but to empirically determine through pilots which types of commands, images, texts, and game scenarios are more familiar to the target population. For example, regarding game controls, after some pilots in MINWii, the WiiPistol replaced the WiiMote control, because the action of pressing a button (to reach the target piano key) was more intuitive than pointing and clicking. Similarly, the touch screen (in Kitchen and Cooking) and the Microsoft^®^ Kinect^TM^ (in X-Torp) were easily employed by the target players, while joysticks and keyboards were avoided.

Another example is represented by X-Torp user-interface, which employed images explicitly associated to the game elements (e.g., a heart to represent life, a fuel can to represent energy) instead of the standard VG color codes (red for life, blue for energy). Also, symbols commonly employed in the menu (e.g., “X” to close a window) were replaced by short text messages (“Close”).

### Explicit Control

Explicit control (**Table 1**) refers to the system capability to respond only to explicit actions of the player without ambiguities or mistakes (e.g., due interference between different command modes).

### Error Management

Error management (**Table 1**) refers to the system capability to promptly detect and fix user errors and mistakes. Concerning these two last “technical” criteria, we believe that the recommendations of Bastien and Scapin (1992) are directly applicable in the framework of the SG for our target players.

## Perspectives on how to Enrich the Ergonomic Criteria for SG

### The Game Rules: The Ninth Criterion

As Bastien and Scapin’s ergonomic criteria are more oriented toward ICT for office automation software, they did not incorporate specific game-related components. For SG, we propose to integrate an additional ergonomic criterion based on a key element of gameplay: the game rules (whose goal is to amuse and entertain), defined through game objectives and means (and/or constraints) to reach them. The game rules criterion sets game’s basic principles, which helps defining the game type, and several motivating aspects of VG, like game scenario, rewarding system or difficulty management. Hence, this criterion would allow the game designer to explicitly address two important game requirements: (1) staying in the VG “mental contest, played… for amusement” mode (Zyda, 2005), despite the constraint posed by the implementation of the pedagogic scenario into the game scenario (Alvarez, 2007); and (2) as it happens when selecting a classical cognitive stimulation task (in which objectives and means to reach them are predefined), choosing a game type (with its own rules) based on the cognitive stimulations to be proposed to the target players.

Many VG studies are now attempting to associate different VG types to specific cognitive stimulations (Powers et al., 2013; Toril et al., 2014). However, they rely on a general game classification that is constantly expanding and becoming more complex (primary game type: action, adventure, role, puzzle, strategy, drive, etc.; game subtype: first person shooter, real time strategy, brain, etc.). The large number of game categories makes it difficult to establish specific links between game types and stimulated cognitive functions. As a result, neuroscience researchers generally classify VG into three broad but reductive categories: action, non-action, and puzzle. Among the several classifications established by researchers in the field of VG (see Mader, 2015), there is one based on the rules of gameplay: the “Gameplay Bricks” (Djaouti et al., 2008).

### The Games Rules Criterion from the “Gameplay Bricks” Model

The “Gameplay bricks” model (Djaouti et al., 2008) states that a game challenge (“Metabric gameplay”) is defined by the combination of a goal (“brick game”) and the means to achieve this goal (“brick play”; see **Table 1**). Based on the review of a large number of VG for entertainment, this model embeds 4 goals: “AVOID”, “DESTROY”, “MATCH” and “CREATE”; and six ways to reach them: “SHOOT”, “MANAGE”, “MOVE”, “RANDOMIZE”, “WRITE” and “SELECT” (see **Table 2**). In total, the model includes 24 game challenges (“metabric gameplay”), which are 24 categories of entertainment-related rules. A VG/SG can thus embed a single challenge or combine simultaneously several challenges. Game type will depend on challenge combinations.

**Table 2:** “Gameplay Bricks” model (Djaouti et al., 2008).

Bricks “Play”		Bricks “Game”	
**Representing the means** This brick invites the user to…		**Representing the goals** This brick invites the user to…	

SHOOT	Hit one or more distant targets	AVOID	Avoid elements, obstacles, enemies or opponents
MANAGE	Manage resources to reach precise objectives	DESTROY	Destroy elements or enemies
MOVE	Drive/lead/take the control of an element or a character	MATCH	Maintain one or more elements in a place, or maintain a defined equilibrium status
RANDOMIZE	Generate a random value	CREATE	Assemble, build, create elements, color, draw from patterns, or predefined brushes; it appeals to the creativity of the user
WRITE	Enter an alphanumeric string as a response, or trigger a game function		
SELECT	Select an item on the screen		

*On the top left, the six “bricks play” (means); on the top right, the four “bricks game” (goals); Each metabrick (gameplay challenge) derived from the combination of one play brick (mean) and one game brick (goal). Hence, it exists 24 metabricks, which reflect 24 possible game challenges.*

For instance, X-Torp integrates three challenges: (1) “Move” to “Match” island; (2) “Move” to “avoid” to be killed; and (3) “Shoot” to “destroy” enemies. However, we recommend for people with MCI or AD not to multiply the challenges. The pedagogical mediator had an indispensable role in determining the usability of X-Torp, in particular to prevent the player from sticking only on one (or two) of the three challenges. Also, due to apathy, we recommend to limit the challenges including as a means of action the “WRITE” Brick (**Table 2**).

Integrating the “Gameplay Bricks” model in the SG ergonomic criteria can open at least three interesting perspectives. First, it would be interesting to look for correspondences between specific challenges and the cognitive stimulations that they can induce starting from the current literature, which is very consistent (Powers et al., 2013; Toril et al., 2014). SG designers would thus be able to choose which game challenges to embed in their SG based on the cognitive functions they are willing to stimulate. Second, it would be interesting to think about extending the “Gameplay Bricks” to multi-player challenges, to further guide game designers wishing to design SG including cooperative challenges (social VG) to improve social engagement. Indeed, the “Gameplay Bricks” focuses only on VG playing in solo mode. Finally, it would be interesting to include in this model a “command mode” component, due to the influence of different control modes on cognitive stimulation (exergames vs. sedentary games).

## Conclusion

The aim of this paper was to present recommendations and perspectives helping professionals involved in the design of SG for the stimulation of people with MCI and ND. Nine ergonomic criteria were presented: eight derived from the work of Bastien and Scapin (1992), and one from the work of Djaouti et al. (2008). We made recommendations starting from scientific papers published on SG for people with MCI or AD up to the moderate stage (Benveniste et al., 2012; Manera et al., 2015; Ben-Sadoun, 2016; Ben-Sadoun et al., 2016). Finally, we added some specific ergonomic recommendations to help researchers and engineers avoiding fatal mistakes, which could significantly delay the final and stable SG version. Agreeing with Karwowski (2006), the compatibility criterion seems to us the most relevant, as it highlights the need to customize the game to the target users, that is to make ergonomic choices in functions of the users, to verify through initial pilots that the SG is a “game” and “usable”.

## Author Contributions

All authors listed have made a substantial, direct and intellectual contribution to the work, and approved it for publication.

## Conflict of Interest Statement

The authors declare that the research was conducted in the absence of any commercial or financial relationships that could be construed as a potential conflict of interest.
